# Conjugation with gum acacia improved the ability of whey protein isolate-(−)-epigallocatechin-3-gallate covalent complexes to stabilize β-carotene emulsions

**DOI:** 10.1016/j.fochx.2025.103339

**Published:** 2025-11-29

**Authors:** Weijun Chen, Hui Hou, Jiaxin Han, Xinhui Wang, Yuncheng Li, Fanbing Meng, Donghong Liu, Xiaoying Guo

**Affiliations:** a College of Food and Biological Engineering, Meat Processing Key Laboratory of Sichuan Province, Chengdu University, Chengdu 610106, China; b College of Biosystems Engineering and Food Science, Fuli Institute of Food Science, Zhejiang University, Hangzhou 310058, China; c Sichuan Kelun Pharmaceutical Co. Ltd., Chengdu 610599, China

**Keywords:** Ternary conjugate complex, β-carotene emulsion, Physicochemical stability, Bioaccessibility

## Abstract

A ternary covalent complex was prepared by conjugating whey protein isolate (WPI)-(−)-epigallocatechin-3-gallate (EGCG) covalent complex with gum acacia (GA). A blue shift in the amide A band and decreased intensity of amide bands in the Fourier transform infrared spectroscopy spectra, and an increase in molecular size confirmed the covalent linkage. It was utilized to stabilize β-carotene emulsion. The freeze–thaw, ionic strength, thermal and pH stability of emulsions were enhanced by conjugation with GA. Compared with WPI, the WPI-EGCG covalent complex and its mixture with GA, the ternary covalent complex used as an emulsifier more effectively inhibited the degradation of encapsulated β-carotene under ultraviolet light irradiation and thermal treatment. The stability of the emulsion during simulated gastrointestinal digestion and the in vitro bioaccessibility of BC also significantly (*p* < 0.05) increased after conjugation with GA. These findings could provide insights into the design of promising and effective food-grade emulsion delivery systems.

## Highlights

• Ternary covalent complex was prepared by conjugation WPI-EGCG conjugate with GA.
• The ternary covalent complex was used to stabilize β-carotene emulsion.
• Physical and digestive stability of emulsion were improved by conjugation.
• Stability and in vitro bioaccessibility of β-carotene was enhanced by conjugation.

## 1 Introduction

β-Carotene (BC) is a natural food coloring agent with anti-oxidant, anti-cancer, anti-cardiovascular and other potential health benefits (Habtegebriel et al., 2024). However, the conjugated double bonds in the molecular structure of BC makes it prone to thermal, light and oxidative degradation (Tufail et al., 2024). The low solubility and instability greatly limit its application in the food industries (Fu et al., 2025). In order to overcome these limitations, various encapsulation systems such as liposomes, nanoparticles, lipid carriers, hydrogels, emulsions have been developed for BC delivery (Vieira & Conte-Junior, 2024). Among these, oil-in-water emulsions are one of the most widely used approaches due to their relatively simple preparation, high encapsulation efficiency, and good biocompatibility (Molet-Rodríguez et al., 2025).

Due to its excellent emulsification performance and high biocompatibility, whey protein isolate (WPI) is often used as an emulsifier to stabilize emulsions (Gao & Qiu, 2025). However, emulsions stabilized by individual proteins easily aggregate under adverse environmental conditions (Li et al., 2025). In addition, the encapsulated bioactive compounds also easily degrade under the effects of light, oxidants, transition metal ions, heat, and other factors (Jakobek, 2015). Thus, strategies are needed to enhance the emulsifying properties of protein.

Proteins, polysaccharides, and polyphenols are nutrients and bioactive substances that often coexist in food systems. Their interaction has become a hot research topic in food science. The interaction of proteins with polysaccharides or polyphenols can occur through noncovalent interactions (e.g., hydrogen bonds, hydrophobic interactions, electrostatic interactions, etc.) and covalent binding, both of which can affect the proteins' structural and functional properties (Cao et al., 2025; Zhang, Wang, Li, et al., 2024). Previous investigations have revealed that protein–polysaccharide/polyphenol noncovalent complexes are sensitive to environmental conditions (e.g., pH, ionic strength, temperature, etc.), as noncovalent interactions are reversible, whereas covalent interactions can lead to the formation of more stable protein–polysaccharide/polyphenol complexes (Wang et al., 2025; Li, Yuan, et al., 2024). Current studies have demonstrated that conjugation with polysaccharides or polyphenols could increase the ability of proteins to stabilize emulsions. It might be mainly due to the steric hindrance provided by the covalent bonded polysaccharides and the antioxidant effect provided by covalently bonded polyphenols (Kouravand et al., 2024; Liu et al., 2025).

Gum acacia, a natural branched heteropolysaccharide, is commonly used as a stabilizer to stabilize emulsions in the food industry (Gavahian et al., 2018). Our previous study revealed that BC emulsions stabilized by WPI-GA covalent complexes presented better physiochemical stability and higher in vitro bioaccessibility than those stabilized by WPI (Chen et al., 2022). (−)-Epigallocatechin-3-gallate (EGCG), the most prevalent and bioactive polyphenols in green tea, is usually used to improve the antioxidant ability of proteins via covalent interactions. Compared to WPI, the emulsifier WPI-EGCG covalent complex (WPI-EGCG con) improved the stability of encapsulated BC (Chen et al., 2023). Similar trend was reported by Qin et al. (2021) about the storage and gastrointestinal passage viability of *Lactobacillus Plantarum* encapsulation with pickering high internal phase emulsions stabilized with WPI-EGCG covalent conjugate nanoparticles. Wang et al. (2019) reported that the glycated WPI-EGCG nanocomplex-stabilized emulsion had greater stability than the WPI-EGCG nanocomplex. Gao and Qiu (2025) also reported that Pickering emulsions stabilized by EGCG enhanced WPI-GA complex showed good stability. These researches proved that the conjugations of GA or EGCG are effective methods to enhance the ability of WPI in stabilizing emulsions.

The applications of protein–polysaccharide/polyphenol covalent complexes in stabilizing emulsions have been extensively researched. However, most of these methods are based on binary covalent complexes, and few reports have focused on ternary covalent complexes. Ke and Li (2024) indicated that soybean protein isolate (SPI)-soluble soybean polysaccharide-EGCG ternary covalent complex exhibited excellent antioxidant capacity and emulsifying property, making it suitable for emulsion delivery systems. Similarly, another study demonstrated that an SPI-β-glucan-myricetin ternary covalent complex, when used to stabilize a BC emulsion, possessed better stability, emulsifying activity and storage resistance under adverse environmental treatment. The protein–polysaccharide–polyphenol ternary covalent complex has the emulsifying activity of protein, the steric hindrance effect of polysaccharides, and the antioxidant activity of polyphenol. Thus, it has better potential in emulsion delivery systems than binary covalent complexes do (Gu et al., 2023; Ke et al., 2025). Therefore in this study, WPI-EGCG con was conjugated with GA to prepare a ternary covalent complex (WPI-EGCG-GA con), and the ability of WPI-EGCG-GA con to stabilize BC emulsions was evaluated.

## 2 Materials and methods

### 2.1 Chemicals

WPI (>93 %) was provided by Hilmar Ingredients (Hilmar, USA), and EGCG (98 %), BC, and bile extracts were obtained from Yuanye Biotechnology Co., Ltd. (Shanghai, China). GA power, Trolox, and pancreatin (8 × USP) were applied by Aladdin Reagent Co., Ltd. (Shanghai, China). Pepsin was applied by Sangon Biotech Co., Ltd. (Shanghai, China).

### 2.2 Preparation and characterization of ternary covalent complexes

#### 2.2.1 Preparation of ternary covalent complexes

The WPI-EGCG con was prepared by an enzymatic method (Chen et al., 2023). Polyphenol oxidase (>500 units/mg dry weight) was added to the WPI solution (10 mg/ mL in deionized water, containing 0.02 % sodium azide) at 10 U/mL. The solution was adjusted to pH 6.5 and stirred at room temperature for 2 h. EGCG was added (0.35 mM) and reacted for 24 h. Then, 5 μM NaHSO_3_ was added to terminate the reaction. The solution was dialyzed against deionized water using a 3.5 kDa molecular weight cut-off (MWCO) membrane at 4 °C for 48 h, with the dialysate being changed every 6 h, and was subsequently freeze-dried. The obtained WPI-EGCG con power was mixed with GA power at a mass ratio of 1:2 to obtain a WPI-EGCG con and GA mixture (WPI-EGCG + GA mixture). The mixture was reacted at 60 °C for 24 h in a dryer with the relative humidity controlled at 79 % by saturated KBr solution, and subsequently freeze-dried to obtain WPI-EGCG-GA con.

#### 2.2.2 Fourier transform infrared spectroscopy (FTIR) analysis

A sample (2.0 mg) was mixed with an appropriate amount of KBr and pressed into pellets. The sample was then scanned by an IR spectrometer within the range of 4000–400 cm^−1^ at an accumulation of 32 scans. The resolution was set at 4 cm^−1^, and the results were exported using OMNIC software.

#### 2.2.3 High-efficiency exclusion chromatography (HPSEC) analysis

The analysis was conducted using Waters e2695 high-performance liquid chromatography with a TSK Gel 5000 PWXL column and a Waters Ultrahydrogel TM 250 column connected in series. The mobile phase was 0.05 M phosphate-buffered saline (pH 7.0) containing 0.15 M sodium chloride. The flow rate was 0.5 mL/min, and the detection wavelength was 280 nm. The protein concentration was 20.0 mg/mL.

#### 2.2.4 Intrinsic fluorescence (IF) analysis

The analysis was conducted according to the method of Chen et al. (2019). The sample was dissolved in 0.01 M PBS (pH 7.0) to obtain a solution with a protein concentration of 0.25 mg/mL. The excitation wavelength was set at 280 nm, and the emission spectrum was recorded from 300 to 500 nm with both slit widths set at 5 nm.

### 2.3 Preparation of the BC emulsion

The aqueous phases were obtained by dissolving WPI (0.80 g), WPI-EGCG con (0.80 g), WPI-EGCG + GA mix (0.24 g) and WPI-EGCG-GA con (0.24 g) in 100 mL of deionized water (containing 0.02 % (*w*/w) sodium azide as an anti-microbial agent). BC was dissolved in corn oil to obtain the oil phase. Then, 10 mL of the oil phase and 90 mL of the aqueous phase were homogenized at 10000 rpm for 10 min to prepare a coarse emulsion. The coarse emulsion was further treated with a high-pressure microfluidizer at 15000 psi for 5 cycles to prepare the final emulsion. The content of BC in the final emulsion was 0.05 wt%.

### 2.4 Microstructural observation

The emulsion samples on the glass slides were covered with cover slips. An optical microscope (UB102i, Chongqing Aopu Optoelectronic Technology Co., Ltd) equipped with an Axiocam ERc5s camera was used to observe the samples at 400× magnification and capture images.

### 2.5 Particle size, polydispersity index (PDI) and zeta potential (ZP) analysis

After dilution 100-fold to avoid multiple scattering effects, the samples were analyzed via a Nano-ZS particle size analyzer (Malvern, UK) at a fixed scattering angle of 90°. All analyses were performed at 25 °C with 3 repetitions.

### 2.6 Physical stability of the BC emulsion

#### 2.6.1 Freeze–thaw stability

A 6 mL aliquot of the oemulsion was transferred into a plastic centrifuge tube, frozen at −18 °C for 24 h, thawed at 25 °C, and then subjected to particle size measurement.

#### 2.6.2 Ionic strength stability

The emulsion was diluted twofold with sodium chloride solutions of different concentrations, stored in the dark at 25 °C for 24 h, and then analyzed for particle size. The final sodium chloride concentration in the diluted emulsion ranged from 100 to 500 mM.

#### 2.6.3 Thermal stability

The emulsion was heated in boiling water for 30 min, rapidly cooled to room temperature, stored in the dark for 24 h, and subsequently measured for particle size.

#### 2.6.4 pH stability

After the pH was adjusted to 3.0, 5.0, or 7.0, the emulsion samples were stored in the dark at 25 °C for 24 h. The changes in emulsion particle sizes were measured.

### 2.7 Chemical stability of BC

The emulsion samples were diluted 5 times with deionized water and transferred to brown glass bottles. The samples were purged with N_2_, and then stored at 5 °C, 25 °C, 37 °C and 55 °C for 30 days. The changes in BC residues were measured using a UV spectroscopy at 450 nm. The ultraviolet (UV) stability and BC content were analyzed as described by Chen et al. (2022).

### 2.8 Digestive stability and in vitro bioaccessibility

The analysis was conducted as described by Chen et al. (2022). Briefly, the digestion process comprised two consecutive phases:

Simulated gastric digestion (SGD): 20 mL of emulsion was mixed with 20 mL of simulated gastric fluid (SGF, pH 2.5) containing pepsin (3.2 g/L). The mixture was incubated in a shaking water bath at 37 °C for 2 h.

Simulated Intestinal Digestion (SID): The gastric chyme was then adjusted to pH 7.0 and mixed with 40 mL of simulated intestinal fluid (SIF) containing pancreatin (8.0 mg/mL) and bile salts (5.0 mg/mL). The mixture was further incubated at 37 °C for 2 h.

Samples were collected after each phase for particle size and zeta potential analysis. For determining the in vitro bioaccessibility of BC, the final intestinal digesta was centrifuged at 5000*g* for 30 min under 4 °C. The BC content in the aqueous micellar phase (the middle layer) was quantified. The in vitro bioaccessibility was calculated as the percentage of BC transferred to the micellar phase relative to the original amount present in the initial emulsion.

### 2.9 Statistical analysis

Analyses were conducted in three parallel groups. Statistical analysis was performed using SPSS version 22 software to evaluate significant differences (*p* < 0.05).

## 3 Results and discussion

### 3.1 Ternary covalent complex characterization

#### 3.1.1 FTIR analysis

**Fig. 1:**
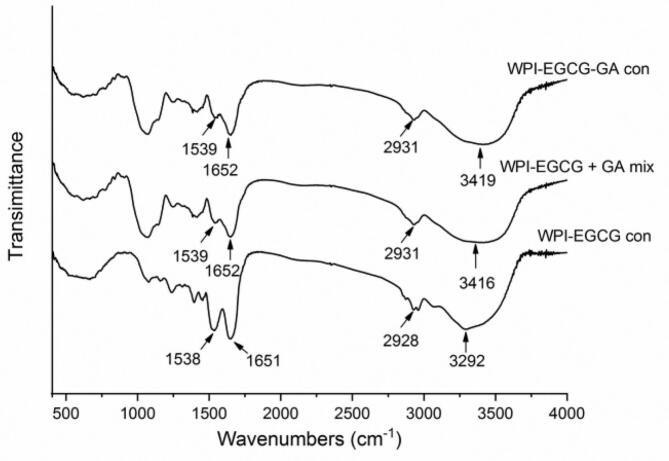
FTIR spectra of WPI-EGCG con, WPI-EGCG + GA mix and WPI-EGCG-GA con.

The consumption of some groups (such as NH_2_) and the formation of others (C=O, C=N, C—N, etc.) during the Maillard reaction may lead to changes in the infrared spectra of proteins (Chen et al., 2019). Fig. 1 reveals that the absorption of WPI-EGCG con at approximately 3292 cm^−1^, 2928 cm^−1^, 1651 cm^−1^and 1538 cm^−1^ are characteristic of the amide A band (N—H stretching), amide B band (C—H stretching), amide I band (80 % C=O stretching), and amide II band (60 % N—H bending and 40 % C—N stretching), respectively (Chen et al., 2023). Compared to the WPI-EGCG + GA mixture, the WPI-EGCG-GA con resulted in a blue shift of 3 cm^−1^ in the absorption peak of the amide A band (from 3416 cm^−1^ to 3419 cm^−1^) and a decrease in the intensity of the amide bands. This indicates a strengthening of the involved chemical bonds or a change in the hydrogen-bonding environment, typically associated with the formation of covalent linkages during the Maillard reaction (Li, Wang, et al., 2024). A previous report revealed that the absorption of amide bands in soy protein isolate decreased with conjugation with dextran via the Maillard reaction (Wang et al., 2025).

#### 3.1.2 HPSEC analysis

**Fig. 2:**
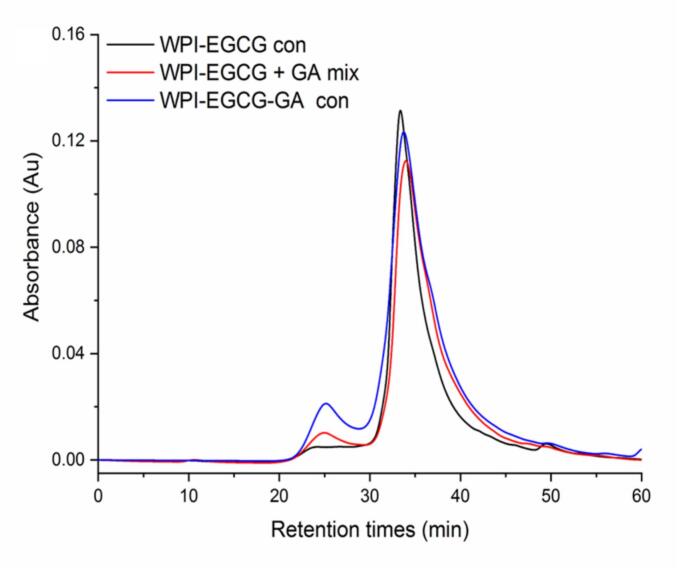
HPSEC elution profiles of WPI-EGCG con, WPI-EGCG + GA mix and WPI-EGCG-GA con.

Fig. 2 shows that only one main elution peak can be observed at approximately 34.00 min for WPI-EGCG con. A new, small elution peak appeared at approximately 25.00 min for the WPI-EGCG + GA mixture. This could be due to the presence of a small amount of protein in GA (Pirestani et al., 2017). Compared to the WPI-EGCG + GA mixture, the new elution peak intensity at approximately 25.00 min of WPI-EGCG-GA con increased. In HPSEC analysis, compounds with higher molecular weights have shorter elution times. This result indicates that new compounds with higher molecular weight were formed. It was because GA was covalently conjugated with WPI-EGCG via the Maillard reaction, leading to the formation of WPI-EGCG-GA con with higher molecular weight. In addition, the elution peak intensity at about 30.00 min for the WPI-EGCG-GA con was stronger than that of the WPI-EGCG con and WPI-EGCG + GA mixture. This might be due to the formation of medium molecular weight covalent conjugates, since both WPI and GA are not single molecular weight compounds.

#### 3.1.3 IF analysis

**Fig. 3:**
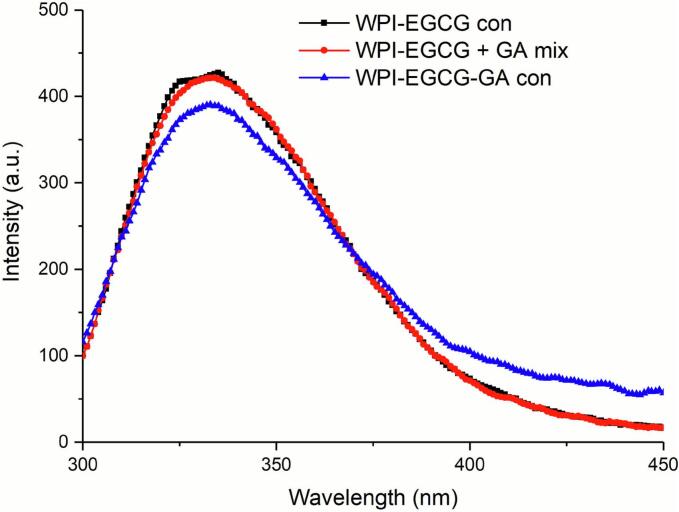
Intrinsic fluorescence spectra of WPI-EGCG con, WPI-EGCG + GA mix and WPI-EGCG-GA con.

Fig. 3 shows that the maximum fluorescence emission wavelength of WPI-EGCG con is around 335 nm, indicating that its fluorophores are located in a “polar” environment (Chen et al., 2019). The fluorescence intensities of both WPI-EGCG + GA mix and WPI-EGCG-GA con are decreased as compared with WPI-EGCG con. This may be due to the shielding effect caused by the polysaccharide chains (Feng et al., 2018). Notably, the WPI-EGCG-GA con exhibited significantly lower fluorescence intensity than that of the WPI-EGCG + GA mix, suggesting that the covalently linked polysaccharide chains provide a stronger shielding effect than the non-covalently linked polysaccharide chains. It may because covalent bonds create a stable and permanent protective layer, while non-covalent bonds form a weaker, reversible layer that can easily fall apart. In addition, the maximum emission intensity of WPI-EGCG-GA con exhibited a slight red shift, indicating an increase in the polarity of its microenvironment (Chen et al., 2019).

### 3.2 BC emulsion characterization

**Fig. 4:**
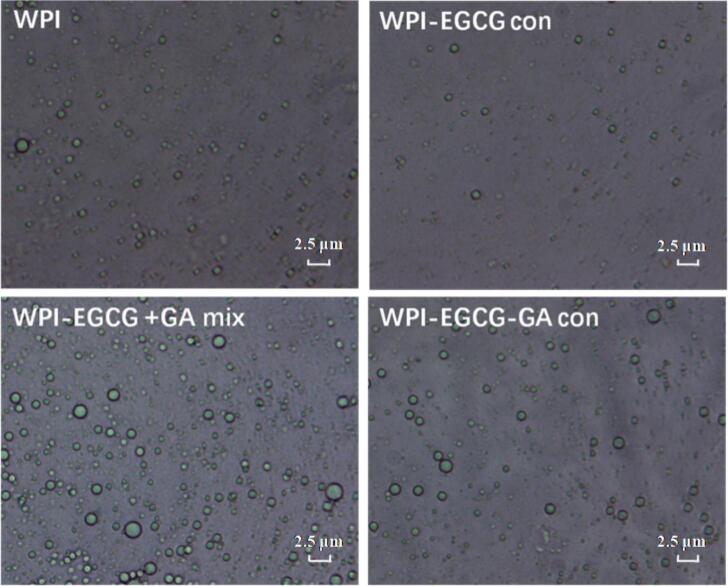
Microstructure of BC emulsions stabilized by WPI, WPI-EGCG con, WPI-EGCG + GA mix and WPI-EGCG-GA con.

Fig. 4 shows that all the emulsion droplets were spherical and distributed evenly. According to Table 1, the WPI-EGCG con-stabilized emulsion had a smaller particle size (221.50 ± 3.73 nm) and greater absolute ZP value (−51.32 ± 1.13 mV) than did the WPI-stabilized emulsion (238.50 ± 4.38 nm and − 47.21 ± 0.41 mV, respectively). The covalent grafting of EGCG decreased the α-helix content and surface hydrophobicity of WPI (Chen et al., 2023). These structure changes may help to increase the “flexibility” of protein molecule, thus reducing its surface tension and isoelectric point at the oil–water interface (Feng et al., 2018). Compared with the WPI-EGCG con-stabilized emulsion, both the WPI-EGCG + GA mix- and WPI-EGCG-GA con-stabilized emulsions had greater particle sizes (304.30 ± 2.97 nm and 277.40 ± 7.07 nm, respectively) and lower absolute ZP values (−45.85 ± 0.21 mV and − 44.70 ± 1.14 mV, respectively). For the WPI-EGCG-GA con-stabilized emulsion, this may be due to the covalently conjugated polysaccharide chain increasing the thickness of the interface layer. With respect to the WPI-EGCG + GA mix-stabilized emulsion, it might be caused by the tendency of polysaccharide molecules to increase the flocculation of droplets via a consumption effect (Iwanaga et al., 2008). The decrease in the absolute ZP value may have resulted from the polysaccharide chain-induced shielding effect and the consumption of amino acids in covalent reactions. However, the absolute ZP values of all emulsions were > 30, which suggests that the resulting electrostatic repulsion generated contributes to the stability of the emulsion system (Lu et al., 2011).

**Table 1:** The particle size, PDI, and ZP of BC emulsions stabilized by WPI, WPI-EGCG con, WPI-EGCG + GA mix and WPI-EGCG-GA con.

Emulsifier	Particle size (nm)	PDI	ZP (mV)
WPI	238.50 ± 4.38^c^	0.194 ± 0.012^a^	−47.21 ± 0.41^b^
WPI-EGCG con	221.50 ± 3.728^d^	0.201 ± 0.016^a^	−51.32 ± 1.13^a^
WPI-EGCG + GA mix	304.30 ± 2.97^a^	0.248 ± 0.024^a^	−45.85 ± 0.21^c^
WPI-EGCG-GA con	277.40 ± 7.07^b^	0.224 ± 0.016^a^	−44.70 ± 1.14^c^

Note: Different letters in the same column indicate significant differences (*p* < 0.05).

### 3.3 Physical stability of the BC emulsion

#### 3.3.1 Freeze–thaw stability

**Fig. 5:**
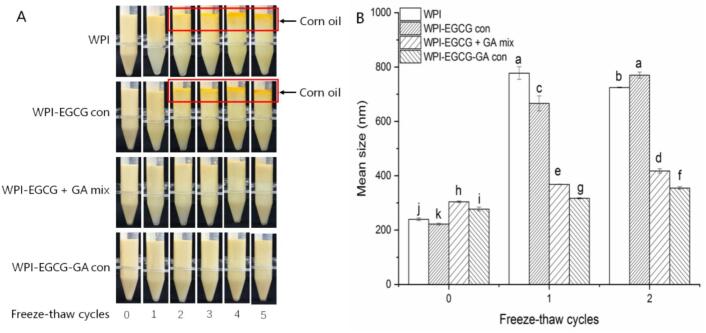
Effect of freeze–thaw treatment on the visual appearance (A) and particle size (B) of emulsions (Different letters (a-k) indicate significant difference at *p* < 0.05).

Freeze–thaw treatment can alter the interaction forces within or between protein molecules, causing a change in their conformation and rendering them unable to maintain interfacial stability (Tan et al., 2011). Fig. 5A shows that the coalescence of emulsions gradually increased with increasing number of freeze–thaw cycles. The oil phase was observed at the upper layer of the WPI- and WPI-EGCG con-stabilized emulsions after 2 treatment cycles, indicating that the emulsions were damaged and that the embedded oil phase had leaked out. The WPI-EGCG + GA mix-stabilized emulsion and WPI-EGCG-GA con-stabilized emulsion showed good freeze–thaw stability, as no oil phase leaked after 5 freeze–thaw cycles. The changes in particle size (Fig. 5B) also confirmed this result. The freeze–thaw treatment-induced increase in particle size was lower when the WPI-EGCG + GA mix and WPI-EGCG-GA con were used as emulsifiers. The WPI-EGCG-GA con-stabilized emulsion presented the lowest rate of increase, indicating that it had the best freeze–thaw stability. This may be because covalently conjugated GA can enhance the spatial repulsion between droplets by increasing the thickness of the interface layer, thus more effectively inhibiting the aggregation of emulsions under adverse conditions (Wang, Zhang, et al., 2024).

#### 3.3.2 Ionic strength stability

**Fig. 6:**
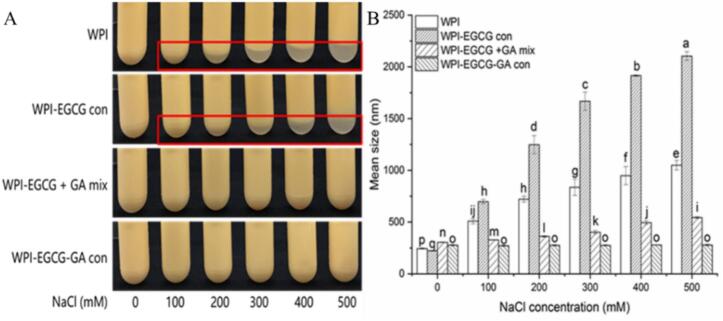
Effect of ionic strength on the visual appearance (A) and particle size (B) of emulsions (Different letters (a-o) indicate significant difference at p < 0.05).

Fig. 6A shows that with increasing NaCl concentration, increased creaming occurred for emulsions prepared with WPI and WPI-EGCG con, whereas no obvious flocculation was observed for those prepared with the WPI-EGCG + GA mix or WPI-EGCG-GA con. Fig. 6B shows that the particle size of WPI-stabilized emulsion increased sharply with increasing NaCl concentration. This was caused by the shielding effect of salt ions on the electrostatic repulsion between droplets (Wang, Huang, et al., 2024). Compared to WPI, WPI-EGCG con as an emulsifier increased the sensitivity of emulsion to salt ions. This might be because EGCG can act as a multidentate ligand to cause cross-linking between proteins when the electrostatic repulsion of the system is low. A much slower increase was observed for the emulsion prepared with the WPI-EGCG + GA mix, indicating that the presence of GA could improve the ionic strength stability of the emulsion. Notably, no significant increases in particle size occurred for emulsion prepared with WPI-EGCG-GA con, indicating the highest ionic strength stability. It was consisted with the report of Zhang, Wang, Liu, et al. (2024) about walnut oil emulsion stabilized by walnut protein isolate-GA. The thicker interface layer formed by the ternary covalent complex (Li et al., 2023) and the repulsive interactions among Maillard complexes might be contributed to the improvement of ionic strength resistance (Zhang, Wang, Liu, et al., 2024).

#### 3.3.3 Thermal stability

**Fig. 7:**
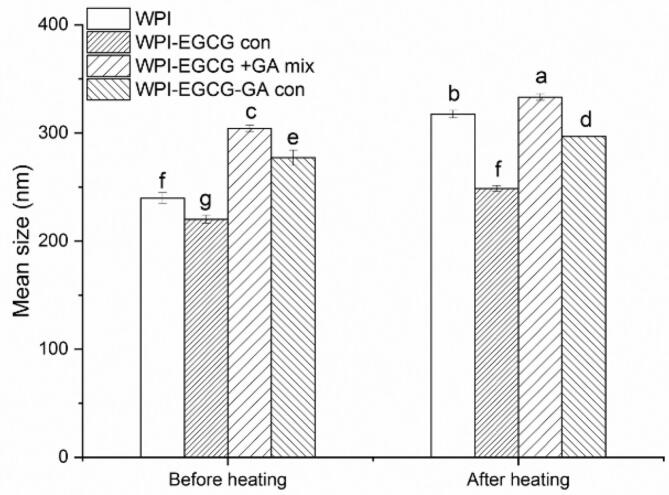
Effect of heating treatment on the particle size of emulsions (Different letters (a-k) indicate significant difference at p < 0.05).

The particle size changes after heat treatment are shown in Fig. 7. It has been reported that high-temperature treatment (> 70 °C, 1 h) could decrease the solubility (a property that is highly related to the emulsifying properties of proteins) of WPI, which may due to the expose of hydrophobic groups and enhancement of the hydrophobic interactions between protein molecules (Chen et al., 2019). This could result the flocculation of emulsion droplets (Zhang, Wu, Liu, et al., 2024). Thus, a significant (*p* < 0.05) increase in particle size was occurred for all the samples after heat treatment. The thermal stability of the emulsions prepared with different emulsifiers decreased in the following order: WPI-EGCG-GA con > WPI-EGCG + GA mix > WPI-EGCG con > WPI. The results showed that the covalent linkage of GA improved the thermal stability of the emulsion as the covalently conjugated polysaccharide chains effectively inhibited heat-induced protein aggregation through steric hindrance (Chen et al., 2024).

#### 3.3.4 pH stability

**Fig. 8:**
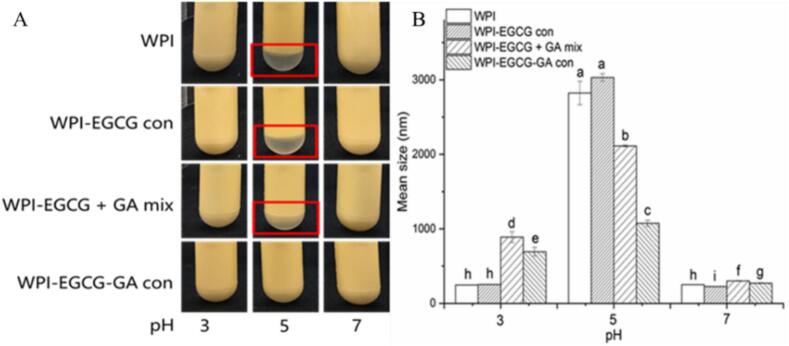
Effect of pH on the visual appearance (A) and particle size (B) of emulsions (Different letters (a–k) indicate significant difference at p < 0.05).

At pH 5.0 (near the isoelectric point of WPI), obvious creaming occurred for emulsions prepared with WPI and WPI-EGCG con, and slight creaming appeared in emulsion prepared with the WPI-EGCG + GA mix, whereas no obvious creaming occurred in emulsion prepared with the WPI-EGCG-GA con (Fig. 8A). Fig. 8B shows that the particle size of the emulsions prepared with WPI and WPI-EGCG con sharply increased to approximately 2800–3000 nm. This might be because the electrostatic repulsion force to maintain the stability of the emulsion was not enough to overcome the van der Waals force and hydrophobic attraction, resulting in droplet aggregation (Tokle et al., 2013). The increase in particle size for the WPI-EGCG + GA mix- and WPI-EGCG-GA con-stabilized emulsions was lower at pH 5.0, indicating that free or covalently bound GA could reduce emulsion aggregation. However, the presence of GA increased the particle size of the emulsion at pH 3.0. This occurred because the negatively charged GA (pKa ∼2.2) neutralizes part of the positively charged WPIs (Chen et al., 2019). Notably, the emulsion prepared with WPI-EGCG-GA con had a smaller particle size than the emulsion prepared with the WPI-EGCG + GA mix at both pH 3.0 and pH 5.0, indicating better pH stability. This may be a result of covalently conjugated polysaccharide chains enhancing the spatial repulsion between emulsion droplets by forming a thicker interface layer (Nooshkam et al., 2023).

### 3.4 Chemical stability of BC

**Fig. 9:**
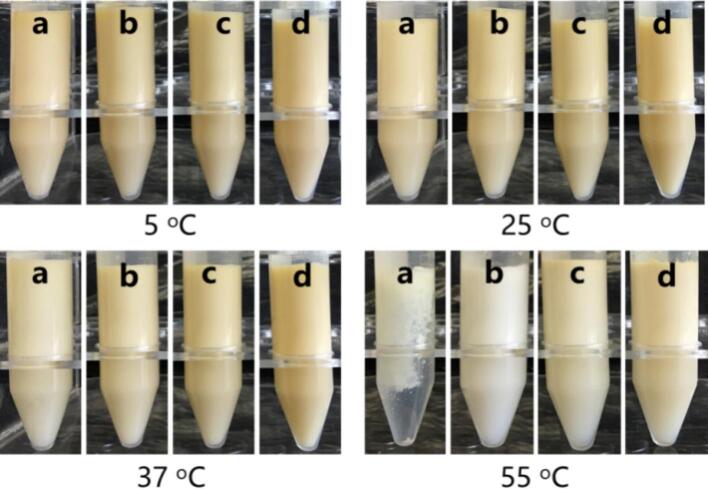
Apparent form of emulsions stabilized by different emulsifiers after storage for 30 days at 5 °C, 25 °C, 37 °C and 55 °C (a: WPI; b: WPI-EGCG con; c: WPI-EGCG + GA mix; d: WPI-EGCG-GA con).

**Fig. 10:**
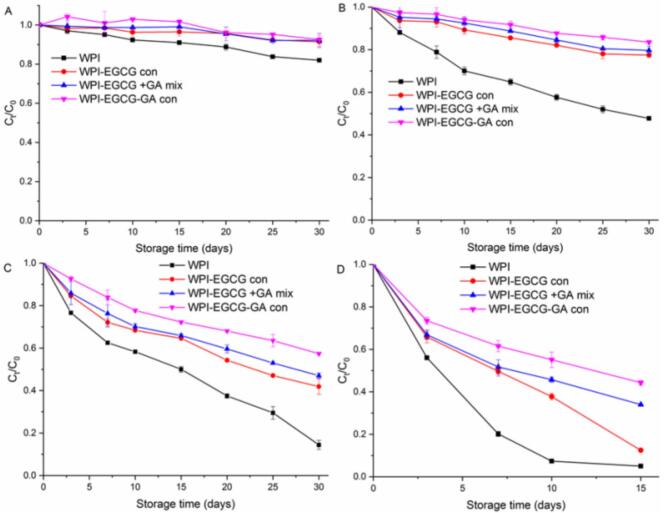
BC retention of emulsions stabilized by different emulsifiers as a function of storage time at 5 °C (A), 25 °C (B), 37 °C (C) and 55 °C (D).

Temperature is one of important factors affecting the degradation rate of BC; thus, the thermal stability of emulsions stabilized by different emulsifiers stored at 5 °C, 25 °C, 37 °C and 55 °C for 30 days was evaluated. Fig. 9 shows the apparent morphology of the emulsions after 30 days of storage. The higher the temperature was, the lighter the color was. Notably, only the WPI-stabilized emulsion was destroyed after 30 days of storage at 55 °C, revealing that its thermal stability was the lowest. One reason for this might be due to the higher storage temperature had a stronger effect on BC degradation. In addition, the thermal denaturation of WPI could decrease the stability of emulsions, thus weaken the protective effect for BC. Fig. 10 shows that BC retention decreased with increasing temperature, which was consistent with the apparent morphology (Fig. 9). The degradation rates of BC in emulsions prepared with different emulsifiers were in the following order: WPI > WPI-EGCG con > WPI-EGCG + GA mix > WPI-EGCG-GA con. For example, after 15 days of storage at 55 °C, the retention rates of BC in these emulsions were 5.02 %, 12.47 %, 33.99 %, and 44.31 %, respectively. In other word, the BC content of WPI-EGCG-GA con-stabilized emulsion was 1.30–8.82 folds as compared with that of other emulsions. This indicates that WPI-EGCG-GA con as emulsifier could enhance the thermal stability of BC more effectively.

**Fig. 11:**
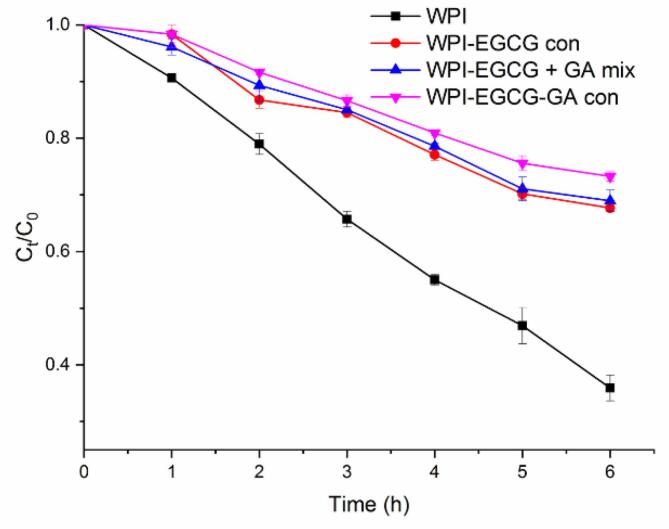
UV stability of BC (C_t_ and C_0_ represent the BC concentration at time t and 0, respectively).

The stability of BC in different emulsions under UV light irradiation was evaluated, and the results are shown in Fig. 11. As peroxide free radicals induced by ultraviolet light can promote the oxidative degradation of BC in emulsions (Liu et al., 2023), the residues of BC decreased gradually with irradiation time. The protective effects of different emulsifiers against BC degradation were in the following order: WPI-EGCG-GA con > WPI-EGCG + GA mix > WPI-EGCG con > WPI. The degradation of BC was effectively inhibited in the WPI-EGCG con-stabilized emulsion compared with the WPI-stabilized emulsion, and the non-covalently and covalently bound GA enhanced the protection of WPI-EGCG con against BC degradation. BC had the highest UV stability in the emulsion stabilized by WPI-EGCG-GA con. After an irradiation of 6 h, the BC content of WPI-EGCG-GA con-stabilized emulsion was 1.06–2.04 folds as compared with that of other emulsions.

**Fig. 12:**
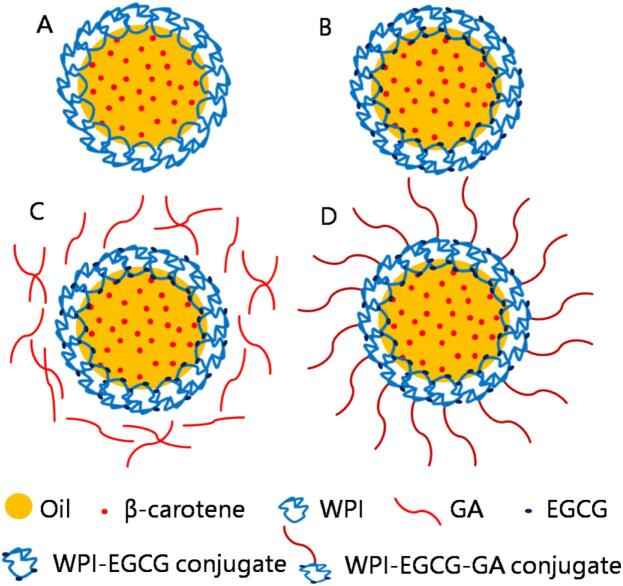
Schematic of BC emulsions stabilized by WPI (A), WPI-EGCG con (B), WPI-EGCG + GA mix (C) and WPI-EGCG-GA con (D).

The above results indicated that WPI-EGCG-GA con was most effective in enhancing the physiochemical stability of BC emulsions. The underlying mechanism could involve the interface layer thickness of the emulsion, the antioxidant, and its physical location in the emulsion system (as shown in Fig. 12). First, a protective layer outside the protein layer was formed by polysaccharides. This could help to inhibit emulsion aggregation under adverse conditions and prevent the diffusion of dissolved oxygen and free radicals at the interlayer. Second, the covalent conjugation of polyphenols endows interface proteins with excellent antioxidant activity, which could act as antioxidants to scavenge free radicals in the system and reduce the oxidative degradation of BC (Zhao et al., 2022). In addition, the free radicals generated by lipid oxidation in emulsions could also result in BC degradation. The oxidation of lipids mainly occurs at the oil-water interface (Shi et al., 2024). Therefore, WPI-EGCG-GA con, which is located at the oil–water interface, can more effectively enhance the stability of BC by scavenging free radicals generated by lipid oxidation.

### 3.5 Digestive stability and in vitro bioaccessibility

#### 3.5.1 Digestive stability

**Fig. 13:**
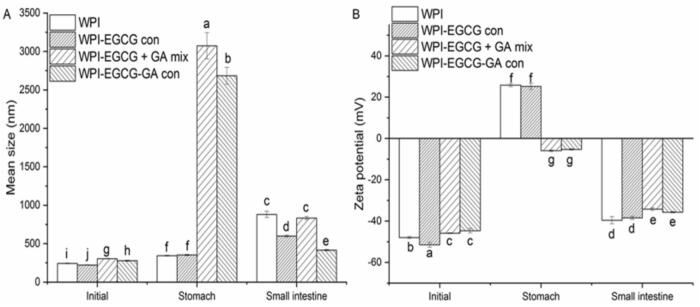
Effect of simulated gastrointestinal digestion on the mean size (A) and ZP (B) of BC emulsions (Different letters (a-i) indicate significant difference at p < 0.05).

Fig. 13A shows that simulated gastric digestion (SGD) significantly (*p* < 0.05) improved the particle size of all samples, suggesting that droplet coalescence and/or aggregation occurred. One reason for this might be that the pH of SGF was lower than the isoelectric point of WPI, which caused the negative charge of the WPI- and WPI-EGCG con-stabilized emulsions to change to a positive charge (Chen et al., 2022). However, due to the negative charge of GA, those prepared with the WPI-EGCG + GA mix and WPI-EGCG-GA con still presented a weak negative charge. In addition, a shielding effect may also be caused by metal ions in SGF. Therefore, the absolute ZP values of all the samples significantly (p < 0.05) decreased (Fig. 13B), which promoted the coalescence and/or aggregation of the droplets. The particle size of the samples increased sharply when GA was present in the emulsions (Fig. 13A). This might be due to the formation of electrostatic complexes between negatively charged GAs and positively charged proteins, which further decreased the electrostatic repulsion force between droplets.

After simulated intestinal digestion (SID), the particle size of the emulsions prepared with WPI and WPI-EGCG con continued to increase (Fig. 13A). This could be attributed to the coalescence and/or aggregation of emulsion droplets resulting from protease-induced interface protein hydrolysis (Liu et al., 2017). Interestingly, the particle sizes of the emulsions stabilized with the WPI-EGCG + GA mix and WPI-EGCG-GA con decreased significantly (*p* < 0.05). This might be a result of the change in pH increasing the absolute value of the ZP, which thereby improved the electrostatic repulsion between droplets. In addition, the protein–polysaccharide electrostatic complex also dissociates at the pH of the SID (Chen et al., 2022). Notably, the particle size of all emulsions was greater than the initial value, which may be due to the following reasons. First, the shielding effect induced by metal ions in SIF decreased the absolute value of the ZP, and thus reduced the electrostatic repulsion between emulsion droplets. Second, the hydrolysis effect of protease could lead to the coalescence and/or aggregation of emulsion droplets. Moreover, some large molecules or colloidal particles produced by lipid hydrolysis may also increase the particle size of the system (Zhang, Wang, Li, et al., 2024). The emulsion stabilized by WPI-EGCG-GA con was the most stable emulsion during the simulated digestion process, possibly because covalently conjugated GA can prevent protein hydrolysis (Liu et al., 2017).

#### 3.5.2 In vitro bioaccessibility of BC

**Fig. 14:**
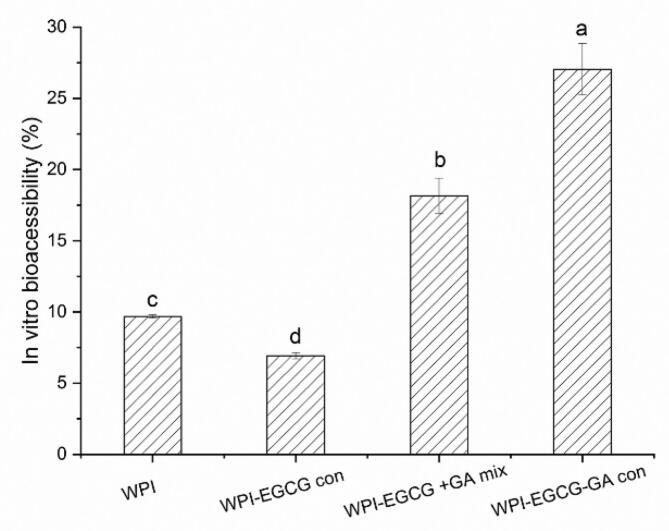
The in vitro bioaccessibility of BC in emulsions stabilized by WPI, WPI-EGCG con, WPI-EGCG + GA mix and WPI-EGCG-GA con (Different letters (a-c) indicate significant difference at p < 0.05).

It is generally believed that water-insoluble functional compounds present only in the micelle phase can be absorbed by the intestinal cells of the human body (Fan et al., 2017). Therefore, the in vitro bioaccessibility of BC was evaluated by measuring its residues in the micelle phase after simulated digestion. Fig. 14 shows that the in vitro bioaccessibility of BC in emulsions prepared with WPI, WPI-EGCG con, WPI-EGCG + GA mix, and WPI-EGCG-GA con were 9.70 %, 6.92 %, 18.15 % and 27.04 %, respectively. These findings revealed that WPI-EGCG-GA con as an emulsifier for stable BC emulsions, can significantly increase (p < 0.05) the transfer of BC to micelles. During the simulated digestion process, the covalently conjugated GA could not only limit emulsion coalescence and/or aggregation caused by the changes of pH and ionic strength, but also prevent the hydrolysis of WPI. Therefore, the emulsion stabilized by WPI-EGCG-GA con has the best digestive stability, which benefits the migration of lipase to the oil.

## 4 Conclusions

EGCG and GA were covalently linked to WPI through enzymatic grafting and dry glycosylation, respectively. The formation of the WPI-EGCG-GA ternary covalent complex was verified by FTIR and HPSEC analyses. All WPI, WPI-EGCG con, WPI-EGCG + GA mix, and WPI-EGCG-GA con as emulsifiers formed stable emulsion systems. Among the studied emulsions, the emulsion stabilized by WPI-EGCG-GA con presented the best freeze–thaw, ionic strength, thermal, pH, and digestive stability. Moreover, the BC in the WPI-EGCG-GA con-stabilized emulsion also had the highest UV light stability, thermal stability and in vitro bioaccessibility. These results indicated that conjugation with GA improved the ability of WPI-EGCG con to stabilize BC emulsion, and could provide insights into the design and application of new antioxidant emulsifiers.

## CRediT authorship contribution statement

**Weijun Chen:** Writing – review & editing, Funding acquisition, Conceptualization. **Hui Hou:** Writing – original draft. **Jiaxin Han:** Data curation. **Xinhui Wang:** Methodology, Conceptualization. **Yuncheng Li:** Writing – review & editing. **Fanbing Meng:** Methodology, Conceptualization. **Donghong Liu:** Methodology, Conceptualization. **Xiaoying Guo:** Writing – review & editing.

## Declaration of competing interest

The authors declare that they have no known competing financial interests or personal relationships that could have appeared to influence the work reported in this paper.

## Data Availability

Data will be made available on request.
